# The Necessity of Implant Removal after Fixation of Thoracolumbar Burst Fractures—A Systematic Review

**DOI:** 10.3390/jcm12062213

**Published:** 2023-03-13

**Authors:** Xing Wang, Xiang-Dong Wu, Yanbin Zhang, Zhenglin Zhu, Jile Jiang, Guanqing Li, Jiacheng Liu, Jiashen Shao, Yuqing Sun

**Affiliations:** 1 Department of Spinal Surgery, Shihezi General Hospital of the Eighth Division, Shihezi 832002, China; 2 Department of Orthopaedic Surgery, Beijing Jishuitan Hospital, Fourth Clinical College of Peking University, National Center for Orthopaedics, Beijing 100035, China; 3 Department of Spine Surgery, Beijing Jishuitan Hospital, Fourth Clinical College of Peking University, National Center for Orthopaedics, Beijing 100035, China; 4 Department of Orthopaedic Surgery, The First Affiliated Hospital of Chongqing Medical University, Chongqing 400016, China; 5 Department of Orthopedics, Beijing Friendship Hospital, Capital Medical University, Beijing 100050, China

**Keywords:** thoracolumbar burst fracture, implant, pedicle screw, removal, kyphosis, complications, pain

## Abstract

Background: Thoracolumbar burst fractures are a common traumatic vertebral fracture in the spine, and pedicle screw fixation has been widely performed as a safe and effective procedure. However, after the stabilization of the thoracolumbar burst fractures, whether or not to remove the pedicle screw implant remains controversial. This review aimed to assess the benefits and risks of pedicle screw instrument removal after fixation of thoracolumbar burst fractures. Methods: Data sources, including PubMed, EMBASE, Cochrane Library, Web of Science, Google Scholar, and Clinical trials.gov, were comprehensively searched. All types of human studies that reported the benefits and risks of implant removal after thoracolumbar burst fractures, were selected for inclusion. Clinical outcomes after implant removal were collected for further evaluation. Results: A total of 4051 papers were retrieved, of which 35 studies were eligible for inclusion in the review, including four case reports, four case series, and 27 observational studies. The possible risks of pedicle screw removal after fixation of thoracolumbar burst fractures include the progression of the kyphotic deformity and surgical complications (e.g., surgical site infection, neurovascular injury, worsening pain, revision surgery), while the potential benefits of pedicle screw removal mainly include improved segmental range of motion and alleviated pain and disability. Therefore, the potential benefits and possible risks should be weighed to support patient-specific clinical decision-making about the removal of pedicle screws after the successful fusion of thoracolumbar burst fractures. Conclusions: There was conflicting evidence regarding the benefits and harms of implant removal after successful fixation of thoracolumbar burst fractures, and the current literature does not support the general recommendation for removal of the pedicle screw instruments, which may expose the patients to unnecessary complications and costs. Both surgeons and patients should be aware of the indications and have appropriate expectations of the benefits and risks of implant removal. The decision to remove the implant or not should be made individually and cautiously by the surgeon in consultation with the patient. Further studies are warranted to clarify this issue. Level of evidence: level 1.

## 1. Introduction

A new spinal fracture occurs every 22 s worldwide [1]. As a mechanical transition junction between the relatively rigid thoracic and the more flexible lumbar spine, the thoracolumbar region is the most common site of fracture to the spine, and burst fractures of the thoracolumbar spine account for approximately 20–50% of such injuries [2,3]. Though common, the management of thoracolumbar burst fractures presents several clinical challenges, which mainly include surgical indications (surgery vs. non-surgery), surgical approach (anterior vs. posterior; traditional open approach vs. minimally invasive percutaneous approach), and surgical options (e.g., short segment fixation vs. long segment fixation, fusion vs. non-fusion) [4,5,6,7,8,9,10,11,12]. In any case, pedicle screw fixation has been well established as a standard procedure for the treatment of unstable thoracolumbar burst fractures that aims to establish immediate stability and rapid restoration of spinal alignment, prevent neurologic deterioration, minimize pain, and protect the spinal cord from further neurological injury [13,14,15,16].

After fracture consolidation has been achieved, there is another considerable controversy related to the pedicle screw instrument removal. So far, several indications have gained wide acceptance for implant removal after spinal surgery, including infection, pedicle screw misplacement, periprosthetic fracture, implant loosening, implant failure, instrumentation protrusion and local irritation, and growth disturbance [17,18,19]. However, the indications, potential benefits, and possible risks for implant removal in successful fracture-healing patients remain controversial [18]. Possible concerns of in situ implants are thought to be reduced range of motion, potential back pain due to mechanical irritation, micromotion, implant prominence and irritation, disc degeneration, facet arthrosis, fretting corrosion, allergic reaction, low-grade infection, stress shielding-related osteopenia, and stress concentration at the adjacent segment [17,18,19,20,21,22,23,24]. Pedicle screw removal might be a beneficial and cost-effective procedure because it can alleviate pain and discomfort, improve the segmental motion angle, restore flexibility, and enhance functional outcomes [25,26]. However, pedicle screw implants should not be considered dispensable when fracture consolidation is present, and implant removal should, by no means, be considered a benign and harmless procedure. On the contrary, implant removal requires a second operative procedure, which is accompanied by risks such as surgical site infection, neurovascular injury, significant loss of segmental kyphosis correction, worsened back pain, and re-fracture [25,26].

To date, there remains a paucity of expert consensus or clinical practice guidelines relating to implant removal after thoracolumbar burst fractures [18]. Thus, we undertook a systematic review to investigate the potential benefit-to-risk ratio and provide up-to-date evidence.

## 2. Materials and Methods

This systematic review was conducted following the recommendation of the *Cochrane Handbook for Systematic Reviews of Interventions* [27] and is reported in compliance with the *Preferred Reporting Items for Systematic Reviews and Meta-Analyses* (PRISMA) guidelines [28].

### 2.1. Data Sources

Electronic databases, including PubMed, EMBASE, Cochrane Library, Web of Science, Google Scholar, and Clinical Trials.gov, were searched from inception to November 2022. Search terms included controlled terms from Medical Subject Headings (MeSH) in PubMed, EMtree in EMBASE.com, corresponding keywords, and free text terms. The search terms included those related to “Thoracolumbar fracture”, “Pedicle screw”, and “Removal”. The complete search strategy is presented in the electronic Supplementary Material Table S1. No language, publication status, or other search restriction was imposed. In addition, we checked the reference lists from all retrieved studies and meta-analyses or systematic reviews already published to ensure that all studies could be identified.

### 2.2. Eligibility Criteria

Published studies were included if they met the following inclusion criteria:

(i) **Participants**: adult patients who underwent internal fixation for thoracolumbar burst fractures;
(ii) **Intervention** and/or **comparison**: removal or retention of the pedicle screw instrument after successful fixation of thoracolumbar burst fractures;
(iii) **Outcomes**: clinical outcomes related to the benefits or harms of implant removal were considered. The primary outcomes were local kyphosis deformity after implant removal and pain intensity after implant removal. Secondary outcomes included improvement of segmental motion angle and removal-related complications;
(iv) **Study type**: All types of studies that reported the benefits and risks of implant removal after thoracolumbar burst fractures were considered for inclusion, including but not limited to case reports and case series, cohort studies, case–control studies, cross-over studies, and randomized controlled trials.

### 2.3. Study Selection

Identified papers from each of the databases were imported into Endnote reference management software X9 (Clarivate Analytics). Two authors independently removed the duplicates, examined the titles and reviewed the abstracts for relevance, and then sorted the remaining records for “inclusion”, “exclusion”, or “potentially relevant”. The full-text articles of eligible records rated “potentially relevant” were obtained, reviewed, and rated independently by the two reviewers. Any discrepancies were resolved by discussion between the authors.

### 2.4. Data Extraction

The data were extracted using a standardized data extraction form and entered into an excel sheet (Excel, Microsoft Corporation, WA, USA). The following study details were extracted where possible from included studies: first author, publication year, region, publication journal, type of study, year of study, sample size, participant demographic details, thoracolumbar fracture level, surgical approach, segmental fixation, time to implant removal, cause of implant removal, and clinical outcomes after implant removal. Data from the research were compared, and disagreements were resolved by consensus among researchers.

### 2.5. Quality Assessment

The Newcastle–Ottawa Scale (NOS) was used to assess the quality of non-randomized studies [29]. The quality of included studies was evaluated in the following three major components: selection of the study group (0–4 points); quality of the adjustment for confounding (0–2 points); and assessment of the outcome of interest in the cohorts (0–3 points). A higher score represented better methodological quality.

### 2.6. Statistical Analysis

Meta-analysis was performed only when there were at least three contrasts available for data synthesis. Risk ratios (RRs) with 95% confidence intervals (CIs) were calculated for dichotomous data, and the mean difference (MD) or standardized mean difference (SMD) along with corresponding 95% CIs were calculated for continuous outcomes. Heterogeneity was assessed using the Cochran Q statistic (*p* < 0.1) and measured with the *I*^2^ statistic. Meta-analyses were conducted using a random-effects model regardless of heterogeneity. Two-sided *p* < 0.05 was considered statistically significant. We used Stata version 15 (Stata Corporation, College Station, TX, USA) for data analyses.

## 3. Results

### 3.1. Study Selection

**Figure 1:**
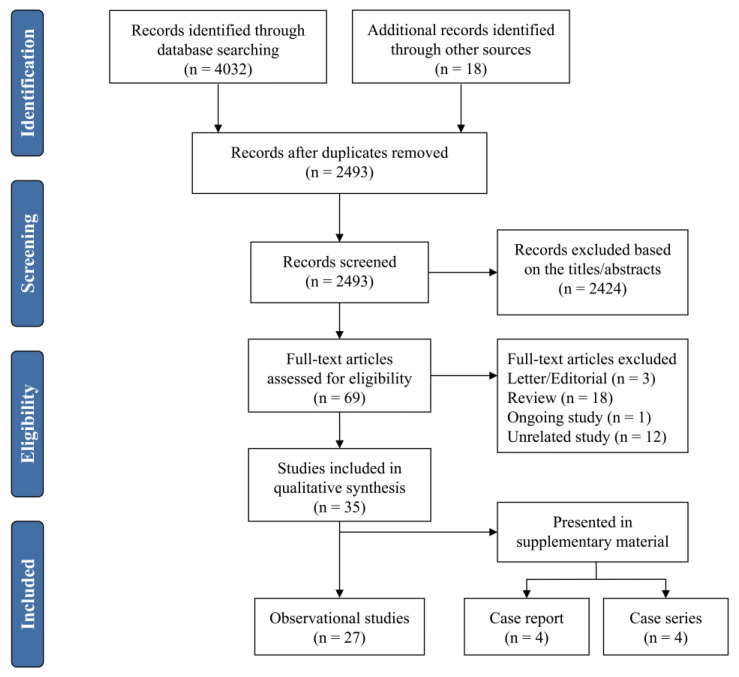
PRISMA flow diagram.

The initial search yielded 4051 records; after removing 1558 duplicates, 2493 articles were screened using the title and/or abstract. Of these, 2424 records were eliminated for being irrelevant to our analysis by screening titles and abstracts. The full texts of the remaining 69 articles were retrieved for further assessment. Finally, 35 studies were included in the systematic review [30,31,32,33,34,35,36,37,38,39,40,41,42,43,44,45,46,47,48,49,50,51,52,53,54,55,56,57,58,59,60,61,62,63,64]. Figure 1 displays a flow diagram that shows the reasons for exclusion at each stage of the selection process.

### 3.2. Study Characteristics

The main characteristics of the included observational studies are presented in Table 1, and the main characteristics of the included case reports and case series are shown in Supplementary Material Table S2. In total, four case reports [30,31,32,33], four case series [34,35,36,37], 21 retrospective cohort studies [38,39,40,41,42,43,44,45,47,48,49,52,53,55,56,57,58,60,61,63,64], three retrospective case-control studies [46,51,62], and three prospective cohort studies [50,54,59] were included in this systematic review. These studies were published between 1997 and 2022 [30,31,32,33,34,35,36,37,38,39,40,41,42,43,44,45,46,47,48,49,50,51,52,53,54,55,56,57,58,59,60,61,62,63,64]. Among the included studies, 25 were from Asia [33,34,35,36,39,40,42,43,44,45,46,47,48,49,50,52,54,55,56,60,61,63,64], six were from Europe [31,32,37,41,51,53], and four from North America [30,38,59,62]. Except for Xu et al. [64], which included patients aged over 65 years, other trials were of adult patients [30,31,32,33,34,35,36,37,38,39,40,41,42,43,44,45,46,47,48,49,50,51,52,53,54,55,56,57,58,59,60,61,62,63]. The fracture level, surgical management, fixation methods, time to implant removal, the reason for implant removal, and duration of follow-up were also quite different among the studies. The more-detailed characteristics of the included observational studies are listed in Table 2, and other detailed characteristics of included case reports and case series are summarized in Supplementary Material Table S3.

### 3.3. Risk of Bias

The pre-planned risk of bias was not assessed during this systematic review. Due to the present lack of high-quality evidence, case reports and case series studies were predetermined to be included to provide related information on our topic. Even if we also included retrospective cohort studies, retrospective case–control studies, and prospective cohort studies, the quality of the observational studies was not assessed due to the inherent biases associated with these study designs and the lack of a control group in many studies.

In addition, the pre-defined meta-analyses were unfeasible due to insufficient data for these clinical outcomes and considerable clinical heterogeneity and variations in outcome measures.

### 3.4. Primary Outcomes

#### 3.4.1. LOCAL Kyphosis Deformity after Implant Removal

Among the 27 observational studies, 20 studies reported varying degrees of sagittal correction loss or local kyphosis deformity, while two studies [43,46] reported no significant kyphosis of the fracture area. In detail, six studies reported average correction loss of less than 5° [39,45,53,55,56,64], nine studies reported 5°–10° average correction loss [40,42,47,48,50,51,54,56,60,63], three studies reported more than 10° average correction loss [38,49,58], and two studies reported 63.9% and 29% local kyphotic deformity after implant removal [57,61].

#### 3.4.2. Pain Intensity after Implant Removal

Of the 27 observational studies, nine [39,41,44,46,49,53,56,59,63] reported significant pain relief after implant removal; of these, four studies [39,41,44,59] reported the decision to remove the pedicle screw instrument due to implant-associated symptoms such as pain or discomfort, while in three studies [46,53,59] the patients were asymptomatic before implant removal. One retrospective cohort study [47] found that 10 of 27 patients had increasing back pain after implant removal, while in another retrospective cohort study [60], 12 of 21 patients reported reduced back pain or discomfort after surgery. In addition, one study [64] found no significant changes after implant removal.

### 3.5. Secondary Outcomes

#### 3.5.1. Improvement of Segmental Motion Angle

Among the observational studies, six [38,44,45,46,47,58] reported improvement after implant removal, three [56,59,63] reported decreased Oswestry Disability Index (ODI) scores, and one [60] reported four of 21 patients had improved range of motion. In contrast, four studies [49,55,62,64] demonstrated no or slight improvement after implant removal but the segmental motion angle was considered to be motionless.

#### 3.5.2. Removal-Related Complications

One case report [30] reported inadvertent screw migration into the retroperitoneal space, while one case series [37] reported that pedicular screws fractured and the threaded parts of the screws were, therefore, left in 1 patient.

For the 27 observational studies, five [38,46,51,52,53] reported wound infection after implant removal, Two studies [39,44] reported vertebral height loss after implant removal, seven [43,45,47,49,50,54,60] reported disc degeneration and progressive loss of injured disc height, and one [40] reported revision surgery after implant removal.

## 4. Discussion

### 4.1. Principal Findings

This systematic review showed that dozens of studies focused on the benefits and risks of implant removal after fixation of thoracolumbar burst fractures, and local kyphosis deformity was the most prevalent and most important sequelae after implant removal. However, some studies further confirmed reduced pain intensity and improved segmental motion angle after implant removal. In addition, implant removal-associated complications were not uncommon.

### 4.2. Comparison with Previous Studies

Kweh et al. published a systematic review and meta-analysis addressing a similar topic [65]. This study eventually included 13 articles for qualitative synthesis and six studies for quantitative synthesis. They found no statistically significant difference in sagittal correction loss between implant retention and removal cohorts, and suggested significantly improved pain intensity and ODI scores. They concluded that planned implant removal results in superior functional outcomes without significant differences in kyphotic angle correction loss compared to implant retention in younger patients with thoracolumbar burst fractures who undergo posterior surgical stabilization. In comparison, we included more types of studies to fully elaborate on this clinical dilemma. Although we did not perform a meta-analysis mainly due to the significant clinical heterogeneity among studies, we found similar benefits but also highlighted the potential risks. We further revealed conflicting evidence regarding the management of thoracolumbar burst fractures. 

### 4.3. Implication for Clinical Practice

Although implant removal accounts for almost one-third of all elective operations in orthopedics, there remains an ongoing debate concerning the justification for such procedures [32]. The thoracolumbar junction is a transitional zone that constitutes the relatively fixed kyphotic thoracic area and the mobile lordotic lumbar region; therefore, it is a vulnerable region for injury. In theory, when natural bone healing and consolidation of fractured vertebrae has occurred, implant removal should allow complete motion segment preservation, but it is hard to decide for the thoracolumbar junction.

#### 4.3.1. Kyphosis Recurrence

Kyphosis recurrence after implant removal is not uncommon (Table 2 and Supplementary Material Table S3). Previous studies have suggested that kyphotic recurrence is inevitable during the medium- to long-term period, regardless of the pedicle screw fixation with or without fusion, and the process of kyphotic recurrence may be accelerated after removal of the pedicle screw instrument, which has been reported in case reports and case series, and some of the observational studies [50,56]. However, there remains a lack of robust clinical evidence and long-term follow-up data, and our systematic review found that currently conflicting data was more present, highlighting this clinical dilemma.

In addition, these studies also investigated the mechanism of sagittal correction loss after implant removal. Some studies [39,44] have implicated that failure to support the anterior spinal column and vertebra collapse after implant removal lead to eventual loss of correction; however, more recent studies [38,46,51,52,53] have found that intervertebral disc collapse and loss of disc height are the main factors contributing to postoperative kyphosis in patients with thoracolumbar burst fracture, no matter with or without vertebroplasty. Patients with incomplete and complete thoracolumbar burst fractures always suffer severely injured endplates and discs, so post-traumatic disc degeneration and height loss when loaded after implant removal are unavoidable. Thus, a mono-segmental fusion is better indicated in cases of expected disc injury to prevent secondary loss of reduction resulting from the collapse of the disc space, especially in younger patients. Removal of the implants may, therefore, not be necessary.

The relatively high incidence of kyphosis recurrence after implant removal may be caused by various factors. The surgical intervention for thoracolumbar burst fracture aims to restore stability, prevent neurological deterioration, attain canal clearance, prevent kyphosis, and provide rapid pain relief. Therefore, sufficient stability is important to avoid postoperative loss of segmental kyphosis correction, regardless of whether fusion is performed. Although the pedicle screw instrument is only to provide temporary fixation of the unstable spine and permanent restoration of spinal stability through achieving a solid fusion as the primary purpose, the pedicle screw instrument may still play an important role in maintaining the reduction, offering rigid fixation, and enhance bony union or fusion after bone healing. A previous study also suggested that the severity of the initial trauma also predicts the loss of correction after implant removal: the more severe the preoperative collapse of the fractured vertebral body is, the higher loss of correction after implant removal has to be expected [51]. In addition, other factors, such as the integrity of the posterior ligamentous complex, are also crucial, and implant removal in patients with non-healing of the posterior ligamentous complex would also induce instability and progressive kyphosis [53,56,66].

#### 4.3.2. Segmental Range of Motion

Improvement of the segmental range of motion has been recognized as one of the major benefits of implant removal, especially in patients who received pedicle screw fixation without fusion. Several previous studies have confirmed the advantages of implant removal for the preservation of segmental motion, which can further alleviate pain and disability [38,44,45,46,47,58] and lead to decreases in the pain intensity score and ODI score [39,41,44,56,59,63]. Therefore, these clinical benefits after implant removal are measurable and demonstrate that a subgroup of patients would benefit from implant removal, especially when there was no disastrous kyphosis deformity recurrence. Nonetheless, we should also realize that the actual mobility of the segment has possible implications—both positive and negative. The improved range of motion of the fractured segment in the thoracolumbar junction would unload the stress on the adjacent segments but put stress on the fractured vertebra and nearby discs. Hence, the improved segmental range of motion also means an unstable status after implant removal, with a potential risk of recurrent kyphosis deformity induced by destabilization after implant removal [37].

### 4.4. Decision-Making

Removing the pedicle screw instrument after posterior fixation of thoracolumbar burst fractures can effectively restore flexibility and relieve pain, but can also result in the progression of kyphosis. Moreover, it is impossible to predict the recurrence of kyphotic deformity before implant removal, and extra revision surgery might be needed later if patients have severe back pain due to severe kyphotic deformity. Thus, careful consideration should be made before removing the implant.

In most symptomatic cases, the patient is the initiator of pedicle screw removal. Many patients with persistent symptoms tend to blame the metallic implants; they often insist on implant removal and believe this will alleviate their symptoms [67,68]. However, in clinical practice, even in patients who have reported implant-related pain, removing the implant does not guarantee pain relief and may be associated with further complications (such as infection, re-fracture, and nerve damage) and worsening pain [31,32,37,41]. Therefore, patients should be notified of indications for implant removal and understand the uncertainty of expected benefits, potential complications, and inherent risks. On the contrary, implant retention would reduce costs and alleviate exposure to further surgery, but patients should also be informed of the possibility of screw breakage.

**Figure 2:**
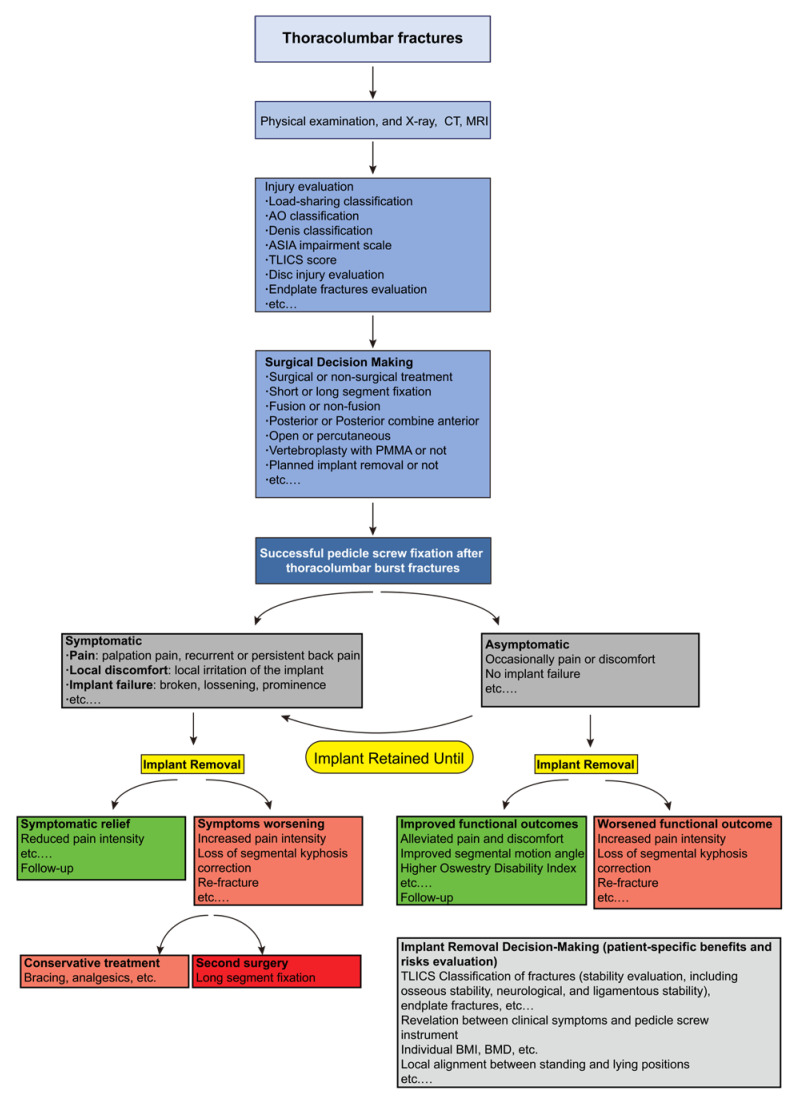
A proposed flow diagram for the management of thoracolumbar burst fractures. Abbreviations: CT, Computed Tomography; MRI, Magnetic Resonance Imaging; ASIA, American Spinal Injury Association; TLICS, Thoracolumbar Injury Classification and Severity; PMMA, Polymethyl Methacrylate; BMI, Body Mass Index; BMD, Bone Mineral Density.

Surgeons are the decision-makers of implant removal [18,67]. The decision of implant removal should be predetermined as early as the initial treatment of the thoracolumbar burst fractures and dynamically adjusted according to the patient’s clinical status (Figure 2). Careful preoperative evaluation and consideration should be made before removing the implant. First of all, surgeons should review details of the primary thoracolumbar burst fractures, such as the mechanism of injury, the morphology, and classification of the burst fractures, and learn about the first surgical management. Second, surgeons need to assess the fusion of the burst fractures, which is critical but challenging, and even intra-operative exploration demonstrates that a solid fusion cannot promise desired outcomes [67]. Next, for symptomatic patients, surgeons should try to figure out to what extent the patient’s pain and discomfort are associated with the pedicle screw instrument, and how much pain relief can be expected from implant removal [69]. For example, postoperative pain may be attributed to instability, root pain, adjacent-level pathology, and factors related to the implant. Very often, the exact cause of post-instrumented pain remains difficult to determine. Finally, communication with patients is essential and crucial than ever before [41]. Patients should be informed thoroughly about the unpredictable outcomes of implant removal to avoid excessively high expectations [41]. Moreover, detailed preoperative evaluation before implant removal is also indispensable. For instance, a CT scan before implant removal would be beneficial for confirmation of posterolateral fusion and preoperative measurement of the bone mineral density of the fractured vertebral body and adjacent vertebral bodies to evaluate the risk of compression fracture after implant removal [31,32]. Based on these careful preoperative clinical evaluations and detailed communication, a decision to remove or retain the implants could be made. The timing of the removal of the implant remains an open question.

### 4.5. Call for Future Studies

The currently available evidence for removing or retaining the pedicle screw instrument in thoracolumbar burst fractures is heterogeneous, limited, and insufficient. Thus, more prospective cohort studies and clinical trials with long-term follow-ups are strongly warranted to provide additional details about the advantages and disadvantages of each option, which would help mitigate the trade-off between the benefits and harms of different treatment options. Second, there is a desperate need to explore the biological mechanisms and clinical determinants of symptomatic and asymptomatic implants, as well as the risk factors and predictive parameters for the recurrence of kyphotic deformity, which will contribute to developing clinical decision rules that may determine which patient subgroup will benefit most from implant removal and which patient subgroup will face more risks [69,70]. Next, future studies should compare the same types of fractures (e.g., incomplete vs. complete burst fractures) when evaluating the outcomes of removing or retaining pedicle screw instruments after thoracolumbar burst fractures, which would help to observe actual clinical outcomes and avoid confusing the effects of fracture types. Additionally, pedicle screw removal is a second surgery performed under general anesthesia, which has substantial economic implications; therefore, a cost-effectiveness analysis should also be performed for policymakers, decision-makers, and other stakeholders [52,69,71].

### 4.6. Limitations

This study has several weaknesses. First, there was substantial clinical heterogeneity among the included studies, including the patient populations (e.g., symptomatic or asymptomatic), the morphology and classification of thoracolumbar burst fractures (e.g., incomplete or complete burst fractures), the severity of injury (e.g., the degree of injury to the discs, the integrity of the posterior ligamentous complex), the treatment strategies of thoracolumbar burst fractures, criteria for implant removal, follow-up duration, etc. These discrepancies reflect the lack of consensus on thoracolumbar burst fractures and compromise the quality of evidence. Second, this study was predetermined to include all kinds of studies, including case reports and case series, which may induce remarkable publication bias, since studies with positive results (e.g., unexpected complications) are more likely to be published in peer-reviewed journals [71]. Third, 25 of 35 included studies were from Asia, mainly from China, Japan, and South Korea, which may also induce bias.

## 5. Conclusions

In conclusion, the removal of implants after successful fusion of thoracolumbar burst fractures may be performed effectively to restore flexibility and relieve pain, but it may also lead to the progression of kyphotic deformity and surgical complications. Both surgeons and patients should be aware of the indications and have appropriate expectations of the benefits and risks of implant removal. There was no robust evidence to support the routine removal of pedicle screw instruments after the successful fusion of thoracolumbar burst fractures, which may expose the patients to unnecessary complications and costs. The potential benefits and possible risks should be weighed to support patient-specific clinical decision-making. Further research is warranted to provide more evidence to clarify this issue.

## Figures and Tables

**Table 1:** Baseline characteristics of the included observational studies.

First Author	Publication Year	Region	Journal	Type of Study	Study Dates	No. of Patients	Age (year)	Gender	Fracture Level	Approach
Knop et al. [38]	2001	USA	Spine	Retrospective Cohort	January 1989–July 1992	76 patients	34 (range 15–63)	26F:30M	Thoracolumbar fractures	Posterior Open
Song et al. [39]	2007	South Korea	Journal of the Korean Orthopaedic Association	Retrospective Cohort	——	58 patients	——	——	Thoracolumbar burst fractures	Posterior approach
Xu et al. [40]	2009	China	Orthopaedic Surgery	Retrospective Cohort	February 1987–June 1995	89 patients	39.1 (range 21–59)	16F:52 M	Thoracolumbar fractures	Posterior approach
Stavridis et al. [41]	2010	Germany	Archives of Orthopaedic and Trauma Surgery	Retrospective Cohort	——	57 patients	46.5 (range 21–84)	28F:29M	Thoracolumbar spine	Posterior approach
Yang et al. [42]	2011	China	Global Spine Journal	Retrospective Cohort	1998–2005	64 patients	42.1 (range 18–70)	24F:40M	Thoracolumbar burst fractures	Posterior Open
Wang et al. [43]	2013	China	European Spine Journal	Retrospective Cohort	July 2007–November 2009	26 patients	39.6 ± 10.3 (range 21–54)	7F:19M	Thoracolumbar burst fractures	Posterior percutaneous
Kim et al. [44]	2014	South Korea	Journal of Korean Neurosurgical Society	Retrospective Cohort	May 2007–January 2011	44 patients	52.5	6F:10M	Thoracolumbar burst fractures	Posterior percutaneous
Ko et al. [45]	2014	South Korea	Journal of Spinal Disorders and Techniques	Retrospective Cohort	September 2003–December 2009	62 patients	38.5 (range 16–54)	29F:31M	Thoracolumbar and lumbar unstable burst fracture	Posterior Open
Jeon et al. [46]	2015	South Korea	Spine	Case–Control	June 2008–October 2011	45 patients	39.7 (range 18–62)	20F:25M	Thoracolumbar burst fractures	Posterior Open
Aono et al. [47]	2016	Japan	Injury	Retrospective Cohort	September 2006–July 2012	27 patients	43 (range 20–66)	8F:19M	Thoracolumbar burst fractures	Posterior Open
Chen et al. [48]	2016	China	International Orthopaedics	Retrospective Cohort	January 2008–December 2013	122 patients	38	49F:73M	Thoracolumbar burst fracture	Posterior Open
Chou et al. [49]	2016	Taiwan	The Bone & Joint Journal	Retrospective Cohort	June 1996–May 2012	69 patients	45.3 ± 10.2 (range 34–56)	25F:44M	burst thoracolumbar or lumbar fracture	Posterior Open
Aono et al. [50]	2017	Japan	The Spine Journal	Prospective Cohort	September 2006–October 2013	62 patients	40 (range13–69)	20F:42M	Thoracolumbar burst fracture	Posterior Open
Hoppe et al. [51]	2017	Switzerland	Global Spine Journal	Retrospective Case–control	2000–2013	59 patients	41.7 ± 15.4	12F:17M	Thoracolumbar fractures	Posterior Open
Lee et al. [52]	2017	South Korea	Spine	Retrospective Cohort	February 2009–May 2012	88 patients	40.2 ± 12.8	23F:22M	Thoracolumbar burst fractures	Posterior Open
Smits et al. [53]	2017	The Netherlands	European Spine Journal	Retrospective Cohort	2003–2015	102 patients	38 (range 18–78)	47F:55M	Thoracolumbar fractures	Posterior open or combined anterior and posterior stabilization
Aono et al. [54]	2019	Japan	Journal of Clinical Neuroscience	Prospective Cohort	September 2006–May 2016	76 patients	40 (range 13–69)	24F:52M	Thoracolumbar burst fractures	Posterior Open
Oh et al. [55]	2019	South Korea	Clinics in Orthopedic Surgery	Retrospective Cohort	March 2011–October 2017	30 patients	41.4 ± 16.0 (range 16–73)	14F:16M	Thoracolumbar fractures	Posterior percutaneous
Chen et al. [56]	2020	China	World Neurosurgery	Retrospective Cohort	February 2008–December 2014	87 patients	41.3 ±8.2 (range 17-60)	28F:56M	Thoracolumbar burst fractures	Posterior Open
Hou et al. [57]	2020	China	Beijing Da Xue Xue Bao Yi Xue Ban	Retrospective Cohort	January 2010–December 2017	144 patients	39.1 ± 13.2	70F:74M	Thoracolumbar burst fractures	Posterior Open
Ko et al. [58]	2020	South Korea	Medicine	Retrospective Cohort	March 2004–January 2007	27 patients	34.8 (range 18–49)	11F:8M	Thoracolumbar burst fractures	Posterior Open
Manson et al. [59]	2020	Canada	Advances in Orthopedics	Prospective Cohort	24-month–8 years	32 patients	38.3 (range 18–61)	8F:24M	Thoracolumbar fractures	Posterior percutaneous
Sasagawa et al. [60]	2021	Japan	Asian Journal of Neurosurgery	Retrospective Cohort	——	24 patients	43.9 ± 12.3 (range 25–64)	4F:20M	Thoracolumbar fractures	Posterior percutaneous
Hirahata et al. [61]	2022	Japan	BMC Musculoskeletal Disorders	Retrospective Cohort	December 2008–June 2016	59 patients	38 (range 17–68)	31F:28M	Thoracolumbar burst fractures	Posterior open
Kenfack et al. [62]	2022	USA	Global Spine Journal	Retrospective Case–control	2012–2017	58 patients	——	15F:43M	Thoracolumbar fractures	Posterior percutaneous
Wu et al. [63]	2022	China	World Neurosurgery	Retrospective Cohort	2018–2020	81 patients	43	21F:29M	Thoracolumbar fractures	Posterior open
Xu et al. [64]	2022	China	Frontiers in Surgery	Retrospective Cohort	August 2011–August 2018	96 patients	69.4 (range 65–77)	51F:45M	Thoracolumbar fractures	Posterior percutaneous or open

Abbreviations: F, female; M, male; ——, Not Reported.

**Table 2:** Reported clinical outcomes in the observational studies.

Study	Fixation	Time to Implant Removal	Pre-Removal	Segmental Motion Angle	Post-Removal Pain	Post-Removal Kyphosis Deformity	Removal Complications	Follow-Up Period
Knop et al. [38] 2001	Short segment fixation	15 (range 7–35) months	——	Improved	——	Average correction loss 10.1°	A late deep wound infection 9 months after removal	25 (range 3–48) months
Song et al. [39] 2007	Fixation with fusion	——	Symptomatic (pain and discomfort)	——	VAS decreased from 6.5 to 3.2	Average correction loss 3.7°	Anterior height of the fractured vertebral body decreased by 1.5% after removal	——
Xu et al. [40] 2009	Short segment fixation	13.2 (range 8–24) months	8 patients with implant failure	——	——	Average correction loss 5.8°	5 patients had local kyphosis of >20°and more back pain, 1 patient underwent revision surgery	8 (range 5–13) years
Stavridis et al. [41] 2010	——	——	Symptomatic (implant-associated pain)	——	VAS from 62 to 48	——	5 of 57 patients (8.8%) had complications, (1 infection, 1 hematoma, 1 transient brachial plexus paresis, 2 immediate postoperative pain)	——
Yang et al. [42] 2011	Short segment fixation without fusion	9–12 months	4 patients with implant failure	——	——	Average correction loss 6.9°	——	——
Wang et al. [43] 2013	Short segment fixation without fusion	9–12 months	——	——	——	No significant kyphosis of the fracture area was diagnosed	The Pfirrmann grade of degenerative discs adjacent to the cranial fractured endplates deteriorated from 2.1 to 3.4 after implant removal	23.5 (15–36) months
Kim et al. [44] 2014	Short segment fixation without fusion	12 months	Symptomatic (pain)	Marked improvement in ROM	Significant pain relief	——	Some vertebral height loss after implant removal	11.8 months
Ko et al. [45] 2014	Short segment fixation without fusion	10 (8–14) months	Selected patients	Improved	——	Average correction loss 1.2° ± 1.63°	Correction loss after removal was due to loss of disk height and/or disk degeneration after implant removal	38 (range 15–79) months
Jeon et al. [46] 2015	Long segment fixation with fusion	18.3 ± 17.6 months	Asymptomatic	From 1.6°± 1.5° to 5.9° ± 4.1°	From 3.8 ± 2.1 to 2.1 ± 1.7	No significant change	3 cases of superficial surgical site infection	2 years
Aono et al. [47] 2016	Short segment fixation without fusion	50 (range 24–84) months	Asymptomatic (except 1 implant failure)	Mean range of motion 8°	10 patients had increasing back pain	Average correction loss 7.5°	Postoperative correction loss occurred due to disc degeneration, especially after implant removal	2 years
Chen et al. [48] 2016	Short segment fixation without fusion	12 months	——	——	——	Average correction loss 6.3°	Kyphosis recurrence 43.4% (53 of 122 patients)	25 months
Chou et al. [49] 2016	Short segment fixation without fusion	10.3 (8-13) months	——	3.8° ± 1.2° (2°–7°)	Significant pain relief, from 6.6 ± 1.6 to 1.7 ± 0.7	Average correction loss 16.6° ± 4.9° (range 6°–26°)	Progressive loss of injured disc height may play an important role in progressive kyphosis	12 months
Aono et al. [50] 2017	Short segment fixation without fusion	12 months	Asymptomatic (except 1 implant failure)	——	——	Average correction loss 9.2° ± 4.0°	Fractured vertebral body was maintained, kyphotic deformity occurred because of a loss of disc height after implant removal	12 months
Hoppe et al. [51] 2017	Short segment fixation with fusion	9.8 ± 4.5 months	Asymptomatic	——	——	Average correction loss 6.0°± 4.2° (range 0°–16°)	——	12.8 (range 11–14) months
Lee et al. [52] 2017	Long segment fixation with fusion	18.7 ± 7.6 months	Asymptomatic	——	——	——	1 superficial wound infection	3 years
Smits et al. [53] 2017	Fixation without fusion	median12 (IQR 10–14) months	Most asymptomatic	——	Majority relief, and minority worse	Average correction loss 4.9°	8 cases of complications (3 superficial wound infection, 2 deep wound infection, 1 instability after removal, 1 bleeding, 1 pneumonia)	>1 year
Aono et al. [54] 2019	Short segment fixation without fusion	12 months	Asymptomatic	——	——	Average correction loss 6.9°	Postoperative kyphotic change was related to disc level, not to the fractured vertebrae	>1 year
Oh et al. [55] 2019	Short segment fixation without fusion	12.8 months	——	Slight improvement after implant removal, mean ROM 4.1° considered to be motionless	——	Average correction loss 3.9° ± 7.3°	Two cases of screw breakage were observed when implants were removed	5.5 months
Chen et al. [56] 2020	Short segment fixation	12 months	——	ODI from 15.9 ± 6.4 to 8.4 ± 4.6	VAS from 2.9 ± 1.3 to 1.2 ± 0.8	Average correction loss 1.5° ± 0.8°	——	>1 year
Hou et al. [57] 2020	Short segment fixation without fusion	12-18 months	Asymptomatic	——	——	Recurrent kyphosis, 92/144 (63.9%)	——	>6 months
Ko et al. [58] 2020	Short segment fixation without fusion	12.2 (range 8–15) months	Asymptomatic	Segmental motion 10.43° ± 3.32°	——	Average correction loss 16.78°	Statistically significant improvement in quality of life over time, with SF-36 56.58 ± 21.56 to 76.73 ± 17.24	>10 year
Manson et al. [59] 2020	Fixation without fusion	16–45 months	Instrumentation prominent or loosening, causing discomfort/pain	Minimal disability after removal, ODI score from 27 to 14	Dropped from moderate to mild/NRS score from 5 to 3	——	——	24 months
Sasagawa et al. [60] 2021	Fixation without fusion	14.4 ± 4.9 (range 5–27) months	——	4 of 21 patients reported improved range of motion	12 of 21 patients reported reduced back pain or discomfort	Average correction loss 9.55°	Disc degeneration happened in 16 of 24 patients	29.1 ± 17.3 (range 3–59) months
Hirahata et al. [61] 2022	Fixation without fusion	16 months	——	——	——	Kyphotic deformity (kyphotic angle >25°) was found in 17 cases (29%)	Loss of correction (kyphotic angle >15°) was found in 35 cases (59%)	15 months
Kenfack et al. [62] 2022	Fixation without fusion	——	——	No significant improvement	——	——	Patient status was not worse after implant removal	——
Wu et al. [63] 2022	Fixation without fusion	8.8-67.1 months	When bone fusion was confirmed on CT	Mean ODI declined significantly	VAS for back pain decreased significantly	Correction loss range 5.0°–8.6°	——	9.1 ± 5.7 months
Xu et al. [64] 2022	Short segment fixation without fusion	16.8 (range 12–34) months	When bony union of the fractured vertebrae was confirmed	ODI from 8.7 ± 10.7 to 8.3 ± 11.0	VAS for back pain from 1.1 ± 1.4 to 1.2 ± 1.6	Cobb angle increased from 9.6° ± 14.1° to 11.4° ± 14.4°	——	33.4 months

Abbreviations: CT, Computed Tomography; NRS, Numeric Rating Scale; ODI, Oswestry Disability Index; ROM, Range of Motion; SF-36, Short Form 36; VAS, Visual Analogue Scale; ——, Not Reported.

## Data Availability

The datasets used and analyzed during the study will be available from the corresponding authors on reasonable request.

## Supplementary Materials

The following supporting information can be downloaded at: https://www.mdpi.com/article/10.3390/jcm12062213/s1, Table S1: Search strategies for primary databases; Table S2: Baseline characteristics of the included case reports and case series; Table S3: The reported clinical outcomes in case reports and case series.

## Abbreviations

ASIA	American Spinal Injury Association
BMD	Bone Mineral Density
BMI	Body Mass Index
CIs	Confidence Intervals
CT	Computed Tomography
MD	Mean Difference
MeSH	Medical Subject Headings
MRI	Magnetic Resonance Imaging
NOS	Newcastle-Ottawa Scale
ODI	Oswestry Disability Index
PMMA	Polymethyl Methacrylate
PRISMA	Preferred Reporting Items for Systematic Reviews and Meta-Analyses
ROM	Range of Motion
RRs	Risk Ratios
SF-36	Short Form 36
SMD	Standardized Mean Difference
TLICS	Thoracolumbar Injury Classification and Severity
VAS	Visual Analogue Scale
