# Supplementary material for: The Necessity of Implant Removal after Fixation of Thoracolumbar Burst Fractures—A Systematic Review
Source: J Clin Med. 2023 Mar 13;12(6):2213. doi: 10.3390/jcm12062213 (PMC10057639; doi:10.3390/jcm12062213)
Supplement: Supplementary file 1 [file jcm-12-02213-s001.zip › Table S1 Search strategies for primary databases.pdf]

Table S1: Search strategies for primary databases

PubMed

Search number	Query	Results
#1	"Spinal Fractures"[Mesh]	17,156
#2	Spinal fracture[Title/Abstract]	851
#3	Spinal fractures[Title/Abstract]	1,547
#4	Spinal burst fracture[Title/Abstract]	15
#5	Spinal burst fractures[Title/Abstract]	22
#6	Thoracolumbar fracture[Title/Abstract]	499
#7	Thoracolumbar fractures[Title/Abstract]	929
#8	Thoracolumbar burst fractures[Title/Abstract]	506
#9	Thoracolumbar burst fracture[Title/Abstract]	272
#10	#1 OR #2 OR #3 OR #4 OR #5 OR #6 OR #7 OR #8 OR #9	18,746
#11	"Thoracic Vertebrae"[Mesh]	22,852
#12	"Lumbar Vertebrae"[Mesh]	59,528
#13	"Vertebrates"[Mesh]	25,248,984
#14	Thoracic vertebrae[Title/Abstract]	1,866
#15	Lumbar vertebrae[Title/Abstract]	4,993
#16	Thoracic[Title/Abstract]	166,622
#17	Lumbar[Title/Abstract]	123,534
#18	Thoracolumbar[Title/Abstract]	11,756
#19	#11 OR #12 OR #13 OR #14 OR #15 OR #16 OR #17 OR #18	301,868
#20	Fracture[Title/Abstract]	206,758
#21	Fractures[Title/Abstract]	182,293
#22	Fracture*[Title/Abstract]	300,860
#23	Burst Fracture[Title/Abstract]	1,048
#24	Burst Fractures[Title/Abstract]	1,176
#25	Burst Fracture*[Title/Abstract]	1,763
#26	#20 OR #21 OR #22 OR #23 OR #24 OR #25	300,860
#27	#19 AND #26	20,479
#28	#10 OR #27	31,695
#29	"Pedicle Screws"[Mesh]	2,744
#30	Instrument[Title/Abstract]	138,828
#31	Pedicle screw[Title/Abstract]	6,741
#32	Pedicle screws[Title/Abstract]	4,211
#33	Instrumentation[Title/Abstract]	41,561
#34	Instrument*[Title/Abstract]	338,944

#35	Implant[Title/Abstract]	147,322
#36	Screw*[Title/Abstract]	52,573
#37	Screw-rod[Title/Abstract]	644
#38	Rod[Title/Abstract]	52,523
#39	Metalware[Title/Abstract]	51
#40	Hardware[Title/Abstract]	28,709
#41	Fixation[Title/Abstract]	168,302
#42	internal-fixation[Title/Abstract]	21,805
#43	#29 OR #30 OR #31 OR #32 OR #33 OR #34 OR #35 OR #36 OR #37 OR #38 OR #39 OR #40 OR #41 OR #42	722,292
#44	remov*[Title/Abstract]	726,845
#45	remove[Title/Abstract]	90,718
#46	removed[Title/Abstract]	239,119
#47	Removal[Title/Abstract]	402,891
#48	#44 OR #45 OR #46 OR #47	726,845
#49	#28 AND #43 AND #47	648

Embase

Search number	Query	Results
#1	'spine fracture'/exp	43744
#2	'spine fracture':ta,ab,kw	1709
#3	'spine fractures':ta,ab,kw	2506
#4	'spinal burst fracture':ta,ab,kw	14
#5	'spinal burst fractures':ta,ab,kw	20
#6	'thoracolumbar fracture':ta,ab,kw	662
#7	'thoracolumbar fractures':ta,ab,kw	1049
#8	'thoracolumbar burst fractures':ta,ab,kw	528
#9	'thoracolumbar burst fracture':ta,ab,kw	300
#10	#1 OR #2 OR #3 OR #4 OR #5 OR #6 OR #7 OR #8 OR #9	44835
#11	'lumbar vertebra'/exp	25732
#12	'thoracic vertebra'/exp	3481
#13	'lumbar vertebrae':ta,ab,kw	6626
#14	'lumbar':ta,ab,kw	160332
#15	'thoracic vertebrae':ta,ab,kw	2427
#16	'thoracic':ta,ab,kw	222222
#17	'thoracolumbar':ta,ab,kw	14266
#18	#11 OR #12 OR #13 OR #14 OR #15 OR #16 OR	381565

	#17	
#19	fracture:ta,ab,kw	237573
#20	fractures:ta,ab,kw	195085
#21	fracture*:ta,ab,kw	326738
#22	'burst fracture':ta,ab,kw	1206
#23	'burst fractures':ta,ab,kw	1300
#24	'burst fracture*':ta,ab,kw	1946
#25	#19 OR #20 OR #21 OR #22 OR #23 OR #24	326738
#26	#18 AND #25	28268
#27	#10 OR #26	61168
#28	'pedicle screw'/exp	11419
#29	'pedicle screw':ta,ab,kw	8444
#30	'pedicle screws':ta,ab,kw	5390
#31	instrument:ta,ab,kw	172611
#32	instrumentation:ta,ab,kw	49643
#33	instrument*:ta,ab,kw	414715
#34	implant:ta,ab,kw	173261
#35	screw*:ta,ab,kw	58413
#36	'screw rod':ta,ab,kw	811
#37	rod:ta,ab,kw	57149
#38	metalware:ta,ab,kw	58
#39	hardware:ta,ab,kw	33139
#40	fixation:ta,ab,kw	176344
#41	'internal fixation':ta,ab,kw	24153
#42	#28 OR #29 OR #30 OR #31 OR #32 OR #33 OR #34 OR #35 OR #36 OR #37 OR #38 OR #39 OR #40 OR #41	837872
#43	removal:ta,ab,kw	475635
#44	removed:ta,ab,kw	323247
#45	remove:ta,ab,kw	115093
#46	remov*:ta,ab,kw	899459
#47	#43 OR #44 OR #45 OR #46	899459
#48	#27 AND #42 AND #47	1048

The Cochrane Library

Search number	Query	Results
#1	MeSH descriptor: [Spinal Fractures] explode all trees	789
#2	(Spinal fracture):ti,ab,kw	2023

#3	(Spinal fractures):ti,ab,kw	1692
#4	(Spinal burst fracture):ti,ab,kw	70
#5	(Spinal burst fractures):ti,ab,kw	73
#6	(Thoracolumbar fracture):ti,ab,kw	336
#7	(Thoracolumbar fractures):ti,ab,kw	306
#8	(Thoracolumbar burst fractures):ti,ab,kw	90
#9	(Thoracolumbar burst fracture):ti,ab,kw	93
#10	#1 or #2 or #3 or #4 or #5 or #6 or #7 or #8 or #9	2648
#11	MeSH descriptor: [Lumbar Vertebrae] explode all trees	2853
#12	MeSH descriptor: [Thoracic Vertebrae] explode all trees	513
#13	(Thoracic vertebrae):ti,ab,kw	1081
#14	(Lumbar vertebrae):ti,ab,kw	4123
#15	(Lumbar):ti,ab,kw	18710
#16	(Thoracic):ti,ab,kw	16710
#17	(Thoracolumbar):ti,ab,kw	951
#18	#11 or #12 or #13 or #14 or #15 or #16 or #17	34596
#19	(Fracture):ti,ab,kw	20992
#20	(Fractures):ti,ab,kw	17438
#21	(Fracture*):ti,ab,kw	26872
#22	(Burst Fracture):ti,ab,kw	123
#23	(Burst Fractures):ti,ab,kw	114
#24	(Burst Fracture*):ti,ab,kw	139
#25	#19 or #20 or #21 or #22 or #23 or #24	26874
#26	#18 and #25	3069
#27	#10 or #26	4728
#28	MeSH descriptor: [Pedicle Screws] explode all trees	81
#29	(Pedicle Screws):ti,ab,kw	447
#30	(Pedicle Screw):ti,ab,kw	673
#31	(Instrument):ti,ab,kw	12984
#32	(Instrumentation):ti,ab,kw	25557
#33	(Instrument*):ti,ab,kw	50552
#34	(Implant):ti,ab,kw	14605
#35	(Screw*):ti,ab,kw	4219
#36	(Screw-rod):ti,ab,kw	32
#37	(Rod):ti,ab,kw	735
#38	(Metalware):ti,ab,kw	6
#39	(Hardware):ti,ab,kw	731
#40	(Fixation):ti,ab,kw	10423

#41	(internal-fixation):ti,ab,kw	1943
#42	#28 or #29 or #30 or #31 or #32 or #33 or #34 or #35 or #36 or #37 or #38 or #39 or #40 or #41	74002
#43	(remov*):ti,ab,kw	40984
#44	(remove):ti,ab,kw	5020
#45	(removed):ti,ab,kw	14037
#46	(Removal):ti,ab,kw	22882
#47	#43 or #44 or #45 or #46	40984
#48	#27 and #42 and #47	50

Web of Science

Search number	Query	Results
#1	Spinal fracture (Topic)	34362
#2	TS=(Spinal fractures)	34362
#3	TS=(Spinal burst fracture)	2131
#4	TS=(Spinal burst fractures)	2131
#5	TS=(Thoracolumbar fracture)	4493
#6	TS=(Thoracolumbar fractures)	4493
#7	TS=(Thoracolumbar burst fractures)	1713
#8	TS=(Thoracolumbar burst fracture)	1713
#9	#1 OR #2 OR #3 OR #4 OR #5 OR #6 OR #7 OR #8	35147
#10	TS=(Lumbar vertebrae)	67790
#11	TS=(Thoracic vertebrae)	26769
#12	TS=(Lumbar)	177577
#13	TS=(Thoracic)	266233
#14	TS=(Thoracolumbar)	11969
#15	#10 OR #11 OR #12 OR #13 OR #14	424743
#16	TS=(Fracture)	1015758
#17	TS=(Fractures)	1015758
#18	TS=(Fracture*)	994312
#19	TS=(Burst Fracture)	5729
#20	TS=(Burst Fractures)	5729
#21	TS=(Burst Fracture*)	5619
#22	#16 OR #17 OR #18 OR #19 OR #20 OR #21	1015863
#23	#22 AND #15	29448
#24	#23 OR #9	50366
#25	TS=(Pedicle Screws)	14126
#26	TS=(Instrument)	1428193

#27	TS=(Pedicle screw)	14126
#28	TS=(Instrumentation)	1709834
#29	TS=(Instrument*)	3038791
#30	TS=(Implant)	627391
#31	TS=(Screw*)	3191244
#32	TS=(Screw-rod)	777386
#33	TS=(Rod)	7576243
#34	TS=(Metalware)	143
#35	TS=(Hardware)	578388
#36	TS=(Fixation)	497739
#37	TS=(internal-fixation)	24676
#38	#25 OR #26 OR #27 OR #28 OR #29 OR #30 OR #31 OR #32 OR #33 OR #34 OR #35 OR #36 OR #37	13487482
#39	TS=(remov*)	4840514
#40	TS=(remove)	3528113
#41	TS=(removed)	3528113
#42	TS=(Removal)	1641282
#43	#39 OR #40 OR #41 OR #42	4840514
#44	#43 AND #38 AND #24	1266

Google Scholar

Search number	Query	Results
#1	("Thoracolumbar burst fracture") AND ("Pedicle Screw" OR "Instrument" OR "Screw-rod" OR "Metalware") AND ("Removal" OR "Removed")	1020
