# Supplementary material for: The Necessity of Implant Removal after Fixation of Thoracolumbar Burst Fractures—A Systematic Review
Source: J Clin Med. 2023 Mar 13;12(6):2213. doi: 10.3390/jcm12062213 (PMC10057639; doi:10.3390/jcm12062213)
Supplement: Supplementary file 1 [file jcm-12-02213-s001.zip › Table S2 Baseline characteristics of the included case reports and case series.pdf]

## Table S2: Baseline characteristics of the included case reports and case series

| First Author | Publish Year | Region | Journal | Type of Study | Study Dates | No. of Patients | Age (year) | Gender | Fracture Level | Approach |
|---|---|---|---|---|---|---|---|---|---|---|
| Vanichkachorn et al.[30] | 1997 | USA | Spine | Case Report | 1996 | 1 case | 40 | M | L1 | Posterior Open |
| Waelchli et al.[31] | 2002 | Switzerland | European Spine Journal | Case Report | 2001 | 2 case | 53 | F | L1 | Posterior Open |
| | | | | | | | 62 | F | L2 | Posterior Open |
| Cappuccio[32] | 2015 | Italy | Journal of Orthopaedics and Traumatology | Case Report | 2014 | 1 case | 29 | M | L2 | Posterior percutaneous |
| Takeda et al.[33] | 2022 | Japan | Surgical Neurology International | Case Report | 2022 | 1 case | 28 | M | L1 | Posterior Open |
| Kim et al.[34] | 2008 | South Korea | Journal of Korean Neurosurgical Society | Case Series | January 2003 ~ May 2005 | 14 cases | 42 (22–67) | 4F : 10M | Thoracolumbar fractures | Posterior approach |
| Wang et al.[35] | 2008 | China | Journal of Neurosurgery: Spine | Case Series | 1999~2003 | 27 cases | 35.4 (19–57) | 8F : 19M | Thoracolumbar burst fractures | Posterior Open |
| Toyone et al.[36] | 2013 | Japan | Spine | Case Series | 2000~2012 | 12 cases | 38 (14–59) | 3F : 9M | Thoracolumbar burst fractures | Posterior approach |
| Axelsson et al.[37] | 2016 | Sweden | Acta Orthopaedica | Case Series | 2007~2011 | 7 cases | 29.4 (19–48) | 4F : 3M | Thoracolumbar fracture | Posterior Open |

Abbreviations: F, female; M, male.
