# Supplementary material for: The Necessity of Implant Removal after Fixation of Thoracolumbar Burst Fractures—A Systematic Review
Source: J Clin Med. 2023 Mar 13;12(6):2213. doi: 10.3390/jcm12062213 (PMC10057639; doi:10.3390/jcm12062213)
Supplement: Supplementary file 1 [file jcm-12-02213-s001.zip › Table S3 The reported clinical outcomes in case reports and case series.pdf]

Table S3: The reported clinical outcomes in case reports and case series

Study	Instrumentation	Time to implant removal	Pre-removal	Segmental Motion Angle	Post-removal Pain	Post-removal Kyphosis Deformity	Removal Complications	Post-removal Follow-up
Vanichkachorn et al.[30] 1997	Short segment fixation with fusion	18 months	Symptomatic (pedicle screw was broken)	——	——	——	Inadvertent screw migration into the retroperitoneal space	——
Waelchli et al.[31] 2002	Short segment fixation with fusion	18 months	Symptomatic (local discomfort presumably due to mechanical irritation)	——	Persistent severe back pain	Kyphosis deformity of 28° on POD 3	Acute T12 compression fracture	——
	Short segment fixation with fusion	12 months	Symptomatic (mild occasional back pain due to putative local irritation of the implant)	——	Increased back pain than preoperative	Kyphosis deformity of 22°	Acute L1 compression fracture	——
Cappuccioet al.[32] 2015	Short segment fixation without fusion	9 months	Symptomatic (local pain, presumably due to mechanical irritation)	——	Completely pain-free 1 year later	No further progression of kyphosis deformity on POD 2	Acute fracture of the superior plate on L1	12 months
Takeda et al.[33] 2022	Short segment fixation with fusion and converted to long segment fixation on POD 22	21 months	Asymptomatic	——	——	Obvious loss of lordosis was observed, and local kyphosis angle increased from 28° to 40°	——	27 months
Kim et al.[34] 2008	Long segment fixation with fusion	15 (range 11-24) months	Symptomatic (recurrent or persistent back pain)	——	Significant pain relief (pain score from 6.4 to 2.6)	5.9° of sagittal correction loss	No major complications	18 (range 12-25) months
Wang et al.[35] 2008	Short segment fixation with fusion	12.5 (range 10–42) months	Asymptomatic (only 2 of 27 patients occasionally need analgesic)	——	——	11.5°±5.4° (2°~22°) of sagittal correction loss	——	2.7 (range 2-4.5) years
Toyone et al.[36] 2013	Short segment fixation without fusion	12 months	Asymptomatic (no failures of posterior instrumentation)	——	——	——	Three reported occasional minimal pain (score 2), and 1 reported moderate pain	>10 years
Axelsson et al.[37]	3 Short/4 Long	18 months	Symptomatic (implant-related	Improved	1 patient residual pain,	1 patient progressive	1 patient the threaded parts	3 months

2016	segment fixation with fusion	back pain)	1 patient unchanged or worse pain	kyphosis deformity 35° and 20°scoliosis; 2 patients had a minor kyphosis of <20°	of the screws were left

Abbreviations: POD, Postoperative day; ——, Not Reported.
